# Nano bio fertilizer capsules for sustainable agriculture

**DOI:** 10.1038/s41598-024-62973-5

**Published:** 2024-06-13

**Authors:** Rinad Hamed, Shehdeh Jodeh, Raed Alkowni

**Affiliations:** 1 Department of Chemistry, An-Najah National University, P. O. Box 7, Nablus, Palestine; 2 Department of Biology and Biotechnology, An-Najah National University, P. O. Box 7, Nablus, Palestine

**Keywords:** Plant sciences, Environmental sciences, Chemistry, Nanoscience and technology

## Abstract

A novel nano bio-fertilizer encapsulation method was developed to crosslink chitosan and alginate with humic acid. These nanocapsules, referred to as (Ch./Alg.HA.NPK) or (Ch./Alg.HA.NPK.PGPRs), were loaded with nanoscale essential agro-nutrients (NPK) and beneficial microorganisms *Pseudomonas Fluorescence abbreviated as* (*P*.Fluorescence). Structural and morphological analyses were conducted using FourierTransform Infrared, Thermogravimetric Analysis, Scanning Electron Microscopy, Malvern Zeta NanoSizer, and Zeta potential. Encapsulation efficiency and water retention were also determined compared to control non-crosslinked nanocapsules. The sustained cumulative release of NPK over 30 days was also investigated to 33.2%, 47.8%, and 68.3%, alternatively. The release mechanism, also assessed through the kinetic module of the Korsemeyer- Peppas Mathematical model, demonstrated superior performance compared to non-crosslinked nanocapsules (chitosan/alginate). These results show the potential of the synthesized nanocapsules for environmentally conscious controlled release of NPK and PGPRs, thereby mitigating environmental impact, enhancing plant growth, and reducing reliance on conventional agrochemical fertilizers**.**

## Introduction

The agricultural sector is a primary source of increasing chemical pollutants via the excessive use of synthetic chemical fertilizers and pesticides, where agrochemical fertilizers are used to promote crop production and soil remediation. In contrast, agro-pesticides manage pathogenic microorganisms to ensure crop health and productivity. Many issues have been reported concerning the problems of excessive and uncontrolled utilization of these agrochemical fertilizers and pesticides, causing harmful pollutants that lead to climate change issues, soil acidification, the decline of soil fertility, nutrient degradation, groundwater pollution, loss of biodiversity, and high energy consumption in the manufacturing process. These chemical pollutants also remain in the environment for generations and can disrupt the hormonal systems of humans and wildlife. Synthetic toxic farms, agrochemical fertilizers, and pesticides are examples of the excessive use of synthetic nitrous oxide (N_2_O) or ammonium nitrate (NH_4_NO_3_) chemical pollutants. Natural denitrification microorganisms reduce the excess of nitrous oxide in the soil to gaseous products, which are then released into the atmosphere, causing significant global warming issues^1^.

Additionally, the biological nitrogen fixation in the soil is affected by the high N_2_, causing the symbiotic natural microbes to be diminished. Furthermore, nitrifying microorganisms utilize the excess ammonium compounds in the soil and produce more nitrate. This high excess nitrate is also utilized by denitrifying microorganism bacterial strains to produce more nitrous oxide (N_2_O), which leaches into fresh, drinkable groundwater resources and leads to more serious water pollution issues and other environmental harms^2^.

The indiscriminate use of synthetic agrochemical fertilizers and pesticides can ultimately cause these environmental issues. Therefore, it has become urgent to develop new alternative agro-based natural techniques and materials to reduce the enormous consumption of agrochemical products^3^. This alternative strategy shall also effectively support plants' innate immunity and bio-stimulate crop production. Great efforts have been made to replace synthetic agrochemical fertilizers with sustainable, eco-friendly natural agrochemical fertilizers and natural agro pesticides, where over the last decade, researchers have shifted towards implementing new nano-enabled agrochemical products based on formulating nano-fertilizer and bio-fertilizer materials, as well on synthesizing natural or synthesized compounds based on formulating eco-friendly materials with different precursor approaches, which acts as antimicrobial agents or plant bio-stimulant or even both of these roles for various agronomical practices^4,5^.

Nano-enabled agrochemicals are classified as nanotechnology techniques in the natural agrochemical field. Nano-enabled agrochemical fertilizers can be divided into macro-, micro-nano, and nano-particulate, based on active nutrients supplied to the plants. Recently, most of these nano-enabled agrochemicals based on nanoparticle formulation can hold the key to developing plant growth within a controlled release of active ingredients and site-specific delivery, with integrated antimicrobial and antioxidant ingredient management^6^. Nano-enabled agrochemicals can also minimize the toxicity of environmental contamination and excessive use of conventional agrochemical fertilizers and pesticides and minimize volatilization, leaching, and run-off agrochemicals to water resources. Moreover, its nano-delivery system can enhance the agrochemical uptake and improve soil and plant stability and solubility, offering a considerable advantage in supporting agriculture and production^7^. Nanoparticulate intelligent delivery system for micronutrient have been developed, evaluand ted and proved to be efficient as compated to conventional fertilizers^8–11^.

Bio- and nano-fertilizers are the main classes of nano-enabled agrochemical fertilizers. These bio- and nano-fertilizer materials mainly apply natural biodegradable, bioavailability, and bio-compatibility polymers in their formulation with nanoscale dimensions ranging from 10 to 1000 nm, depending upon the various green preparation precursors method for preparation, nanosphere, or nanocapsule to be obtained, and bioactive agro ingredients or inoculant microorganisms are dissolved, encapsulated, entrapped, or chemically or physically bound to prepared matric^12^. The encapsulation mechanism of active agrochemical ingredients or microorganisms inoculants can be described as enclosing required particles from solids, droplets of liquids, gases, or inoculant beneficial microorganisms into an inert polymeric shell, which isolates and protects it from the external environment's stress and deteriorative reactions and regulates the release and diffusion of incorporated compounds in a controlled and gradual manner at the nanoscale with a rate required for a specified treatment to meet the plants' needs, offering significant agriculture and environmental advantages^13^. Thus, these new nanoformulation materials make them ideal as emerging alternative conventional synthetic chemical fertilizers for retaining safe, reliable, clean, non-toxic, low-cost, and eco-friendly, with sustained-release nanoparticles in a controlled process that are highly efficient for plant fertilization and regulation, and help us eliminate environmental contamination and eutrophication^14^.

A variety of natural polymers have been used in nano-enabled agrochemical fertilizers to entrap hydrophilic, hydrophilic substances, or inoculant microorganisms, including proteins, polysaccharides, synthetic and natural polymers, lipids, dendrimers, and peptides. Naturally occurring biopolymers such as chitosan, alginate, starch, and cellulose are considered predominant polysaccharides utilized in agriculture manufacturing for entrapments active bio ingredients and formation of either nano-fertilizer or bio-fertilizers^15^.

Chitosan is considered the most studied biopolymer and has received remarkable attention over recent decades due to its biocompatibility, biodegradability, bioactivity, and non-toxicity. Chitosan is also the most abundant biopolymer after cellulose with distinct properties of bio-adhesion, adsorption enhancer, coating, anti-inflammatory, antimicrobial, antifungal, and antiviral. Furthermore, chitosan consists of many hydroxyl and amino groups along its polymeric chain, making it suitable for ease of chemical modification and functionality. Several function groups can functionalize chitosan^16^. Functionalization can be addition, coupling, grafting, cross-linking, and other synthetic routes in preparing nanoparticles, suspensions, composites, antimicrobial materials, functionalized materials, or (nano) hybrids, where the functionalization of chitosan with other materials can enhance and advance its features for diverse eco-friendly agriculture purposes and applications^7,17,18^.

Many efforts have been encountered in formulating nanoparticles as either nano-fertilizers or bio-fertilizers for altering environmental risks. These nano–and bio-fertilizers have many classifications regarding their synthesis and formulation techniques. Nano capsules are the most abundant methods for encapsulating active agronutrient materials or microorganism inoculants. Also, these nano-capsules mainly consist of chitosan–alginate polymer matrices due to their unique physical and chemical characteristics. Some of these nano capsules have been recently modified and cross-linked with other polymers to advance their mechanical strengths and sustain their steady-state release^14,19^. Still, until now, no research has handled the formulating nano bio-fertilizer cross-linked with humic acid material.

Different studies have emerged on the importance of cross-linking of chitosan–alginate nanocapsules to increase efficiency in controlling the release of trapped active agro-nutrients.

A study by Dhiman et al., explored the chitosan-alginate nano capsules for encapsulating NPK active nutrients in their review. The nanoencapsulation method was found to have advanced the stability and mechanical strength of the formulated nano capsules, facilitating their controlled release mechanism with a prolonged release^20^.

A study by Mesias et al., investigated the encapsulation of NPK in chitosan alginate no capsules cross-linked with calcium chloride and citric acid. The results revealed that the cross-linked effectively protected NPK from fast leaching, volatilization, and degradation, improving its release. The results show high encapsulation efficiency and sustained nutrient release, improving their uptake within plants’ rhizosphere and interactions. These studies highlight the potential cross–linked benefits of chitosan-alginate nanocapsules for encapsulating NPK active agro nutrient at the nanoscale, offering controlled release and sustained nutrient management in agriculture practices^21^.

In a study for bio-fertilizers, Mohsin et al., demonstrated in the review that the application of polysaccharide in the encapsulation of beneficial microorganisms enhanced the survival rate and controlled the release of PGPR microorganisms, promoting their colonization in the rhizosphere and stimulated plant growth. These findings highlight the potential of chitosan-alginate nanocapsules cross-linked with other materials as efficient delivery systems for NPK and PGPRs microorganisms, offering sustainable agriculture strategies^22^.

In this work, it introduces a novel nano biofertilizer (Ch. Alg./HA/NPK/PGPRs) made by cross–linking of chitosan/alginate nanoparticles with humic acid to encapsulate active agro-nutrients NPK and beneficial microorganisms PGPRs (*Pseudomonas Fluorescence*). Indeed, to determine the effect of the humic acid as the cross-linker on improving the nanocapsule encapsulation efficiency, water holding capacity, biodegradability, biocompatibility, and its controlled mechanism release behavior for entrapped materials. This innovative approach aims to address the challenges of controlled nutrient release in agriculture applications, which could pave the way for sustainable agriculture production and minimize the impact of environmental issues.

## Materials and methods

### Materials

All reagents and chemicals used in this study were purchased from Sigma Aldrich and used in the research paper without further purification (Chitosan, Alignate, calcium chloride, glacial actetic acid, sodium hydroxide, Tween20, hydrochloric acid, calcium hydroxide, sodium chloride, ammonium chloride, potassium chloride, Calcium dihydrogen phosphate, trehalose sugar). Distilled water was used for synthesis and antimicrobial procedures.

### Instruments

Fourier Transform Infrared (FT-IR) spectra were acquired in the 4000–500 cm^−1^ range with 64 scans, each 8 cm^−1^ resolution by Thermo Scientific Nicolet IS5 equipped with an ATR sampling device, and adetector of Deuterated Triglycine Sulfate.The Malvern Zeta Sizer NanoSizer instrument was used to analyze particle size and zeta potential of prepared nanoparticles with Malvern and zeta sizer software at (Forschungszentrum Jǘlich) and with detectors of Non- Invasive Back Scatter (NIBS) for size measurements and a dectector of Electrode Assembly for zeta potential measurements.

The Scanning Electron Microscope (SEM) photograph was obtained by Zeiss Sigma 500 SEM JEOL 7400 E Scanning Electron Microscopy, and the samples were gold-plated at (Forschungszentrum Jǘlich). The thermal stability for prepared nanocapsules was measured using a Thermo Gravimetric Analyzer (TGA) (Lenovo V520, China) coupled with an MS- Thermostat GSD3220 (Pfeiffer Vacuum) and measured with Pt crucible in N_2_gas flow (20 ml min^−1^) with a heating rate of 5 °C in the range 25–900 °C. The program was managed by the STAR software v 10.00 (Mettler Toledo). The potassium and phosphorous ions were analyzed using a Thermo I CAP 7000 series ICP spectrometer, and the nitrogen ions were analyzed using a Continuous Flow Analysis (CFU) alliance instrument at (Forschungszentrum Jǘlich).

### Preliminary study for synthesis of nano-bio fertilizer capsules

**Figure 1:**
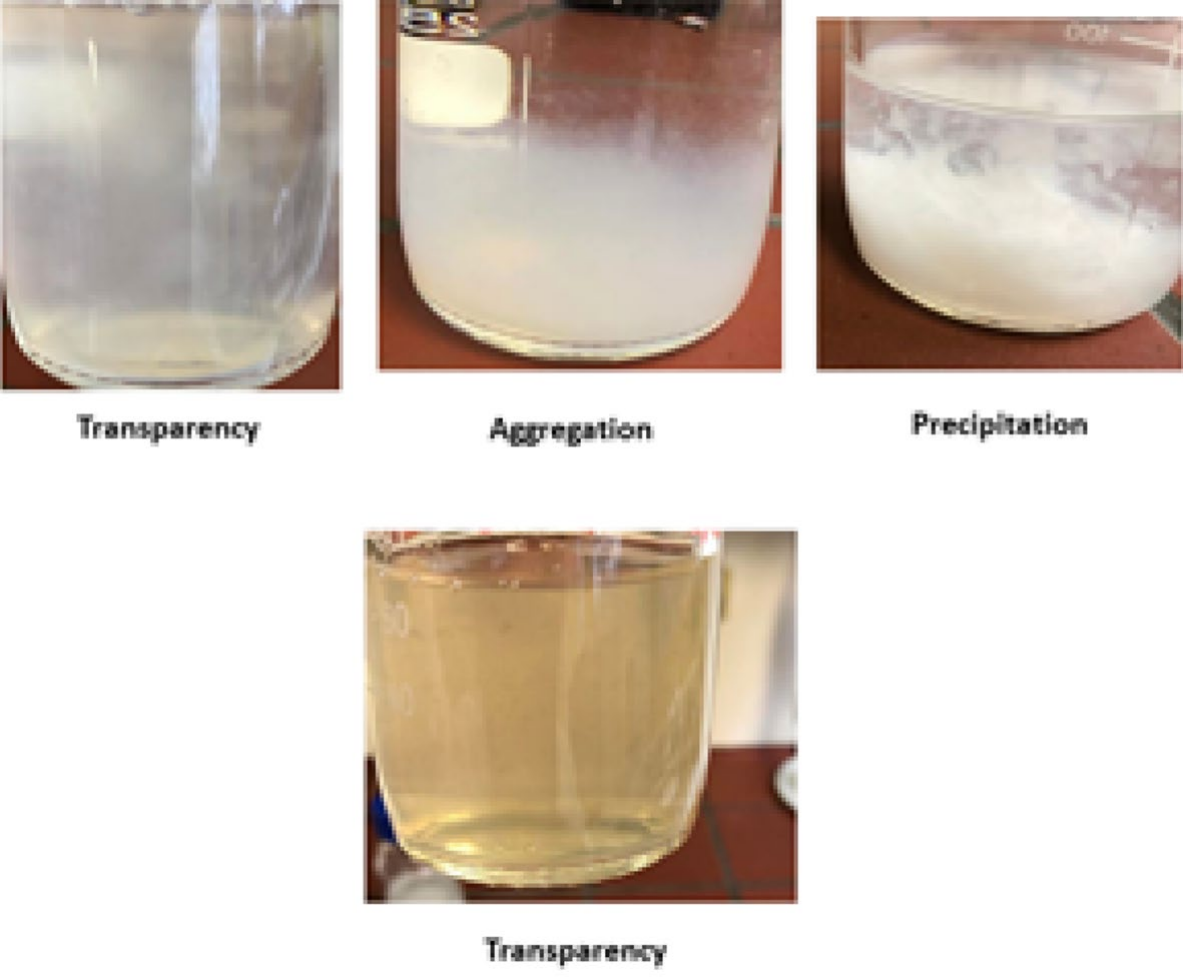
The appearance of prepared nano capsules solutions.

The synthesis approach to obtaining the final nano bio fertilizer capsules was based on preliminary and observational stability studies. All these tests were conducted at varying chitosan, alginate, humic acid, and NPK concentrations until the best base model was identified and determined. The optimized parameters of proper pH value, homogenization time, needle gauge size, equilibration time, stirring time, and calcium chloride concentration were also set to obtain the smallest particle size and highest encapsulation efficiency. The observational stability of nanoparticles was taken as transparency, precipitation, and aggregation of these formulated nanocapsules. Figure 1 describes these functions. The Best stable nanoparticle solution with homogenous nanocapsules was recorded as the translucent solution with the best transparency observation. Meanwhile, the solutions with opaque, transparent, precipitation, or aggregation solutions and the formulated nanoparticles that had settled down to the bottom of the sample solution immediately or overnight were considered unstable solutions with poor nanoparticle stability. All optimized parameters for the synthesis process with the best particle size and encapsulation efficiency were consolidated and extrapolated into the final nano bio-fertilizer formulation^23,24^.

#### Stock solutions preparation

The following stock solutions were prepared according to the previously mentioned protocols with some modifications.

##### Chitosan nano particle solution (0.05% w/v)

A 0.1 g of chitosan material was added to 150 ml of distilled deionized water in a 200 mL volumetric flask and stirred for 30 min. Then, 1 mL of glacial acetic acid was added and allowed to stir for 6 h. After that, the chitosan solution was filtered using vacuum filtration, and its volume was adjusted to 200 mL with the addition of water. Moreover, its pH value was adjusted to 4.6 by adding 6–11 drops of 5 N NaOH to obtain a final chitosan nanoparticle stock solution^23^.

##### Alginate nanoparticle solution (0.06% w/v)

A 0.15 g of sodium alginate was added to 200 mL of distilled deionized water in a 250 mL volumetric flask and stirred for 4 h until fully dissolved. Then, the alginate solution was filtered using vacuum filtration using a qualitative filter paper with 10 µm particle retention. After that, 1.25 mL of Tween 20 was added to the solution and stirred for another 2 h until the solution appeared homogenous. Finally, its volume was adjusted to 250 mL by adding water, and pH was adjusted to 4.9 by adding 5–9 drops of 0.5 N HCL to obtain a final alginate nanoparticle stock solution^23^.

##### Humic nanoparticle solution (1% w/v)

Humic acid was extracted from peat soil by alkaline extraction method^25^. A 20 g of air-dried sieved homogenized peat soil was mixed in a beaker provided with a four-blade 45 pitched blade stirrer having a diameter of 0.4 m with 100 mL of 0.1 M Ca(OH_2_) and 100 mL of 0.1 M NaOH at a rotation speed of 300 rpm for 5 h at ambient room temperature. Then, it was centrifuged at 10,000 rpm for 30 min. The obtained supernatant was acidified with 6 M HCl till a pH adjusted to be one and left overnight. After that, the formed humic acid fraction was filtered using a sintered glass funnel and dried in an oven at 80 °C, resulting in a solid dark humic acid product. Finally, a stock humic acid solution (1% w/v) was prepared by dissolving obtained humic acid in distilled water and filtered using a qualitative filter paper with 10 µm particle retention, then stored in the dark till further use. The obtained humic acid was characterized and compared to the humic content data from the International Humic Substances Society.

##### Selecting and culturing PGPR (*Pseudomonas* fluorescence (PL 5.4))

*Pseudomonas fluorescence* (PL 5.4) bacteria strains were cultured in Tryptic Soy Broth (TSB) media and incubated at 30 °C for 48 h. After growth, these bacterial strains were striated by depletion on Trypticase Soy Agar plates and incubated at 30 °C for 48 h.

For nano bio-fertilizer capsule preparation, precisely 50 mL of *P. Fluorescence* bacterial culture was centrifuged at 3000 rpm for 10 min. The cells were harvested by centrifugation and washed with 0.85% NaCl (w/v) saline solution, then 10 mg of trehalose sugar was added to enhance the stability of the PGPR bacterial strain without contamination and carried to a shaker for 72 h. The last cell is obtained as a log growth phase with a 4 × 10^8^ CFU mL^−1^ cell density. Then, it was preserved for the preparation of nanocapsules.

### Ionotropic gelation of nanocapsules and polyelectrolyte complexation

The ionotropic gelation step includes 1 g of NH_4_Cl, 1.94 g of KCl, 1.62 g ofCa (H_2_PO_4_)_2_ as a source of N, P, and K fertilizers were dissolved into the previously 250 mL with a pH adjusted (4.9) sodium alginate stock solution and stirred for 2 h at ambient room temperature. After that, 15 mL of 0.2% (w/v) calcium chloride solution was added dropwise with an adapted burette syringe through a 20-gauze needle into sodium alginate solution to form pre-gel). Subsequently, the pre-gel was stirred for 90 min under mild agitation and sonicated for three cycles of 5 min. Its temperature was raised to 70 °C, and the 15 mL of 1% (w/v) humic acid was added as a cross-linked and immobilized bacterial strain and allowed to stir at 500 rpm for 2 h. After that, the pre-gel mixture was allowed to cool at room temperature, around 25 °C, and previously prepared bacterial cell pellets were transferred to the pre-gel mixture, mixed thoroughly for another 2 h, and set for the polyelectrolyte complexation step^23,24^.

The polyelectrolyte complexation step involved 200 mL of pH-adjusted (4.6) chitosan stock solution was extruded dropwise with an adapted burette- syringe through a 20-gauze needle into a stirred prepared pre-gel mixture. The resulting nanoparticle solution with homogenous nano capsules was recorded as the translucent solution with the best transparency observation and was stirred for 90 min at room temperature and set down for the lyophilized procedure to obtain a final dried weight of 2.782 g with a yield of 85.6%

On the other hand, the control samples prepared contained chitosan/alginate nano capsules with encapsulating NPK active nutrients, and the weight of dried nano capsules was 2.3075 g with a yield of 71%.

All tested trail samples were weighted after the lyophilization process. The yield analysis was calculated from the weight of dried nano capsules recovered as (W_1_) and divided over the sum of the initial dry weight of starting materials used (W_2_). The yield percentage was determined according to the following equation:

1 $${\text{Yield }}\left( \% \right) \, = \frac{{W_{1} }}{{W_{2} }} \times 100$$

where W_1_ = weight of dried nano capsules recovered. W_2_ = weight of the initial dry weight of nano capsules.

#### Homogenization, purification, and lyophilization of nano bio-fertilizer capsules

The prepared nano bio-fertilizer capsules were homogenized at 10,000 rpm in 5 min to prevent suspension overflow. Then, it was sonicated three times for 5 min each cycle; then, the suspension was allowed to settle and equilibrate overnight to achieve a complete ionotropic gelation polyelectrolyte complexation. Then, the suspended nano capsules were purified through filtration to remove any excess nano capsules, large aggregates, and undissolved NPK materials. Filtered nano capsule materials were transferred into a 250 mL glass-Prex container and sealed with a lid to be frozen overnight at – 20 °C. After the retentate material was frozen, the Pyrex lid was replaced with permeable tissue paper and placed again into a freezer dryer at – 50 °C for 72 h to sublimate all nanoparticle moisture. After that, all prepared samples were removed and weighed out: the control sample (2.3075 g) was white, and the cross-linked sample (2.9148 g) was light brown. All lyophilized trails were stored in an air-tight container in a freezer at – 20 °C until further characterization^23,24^.

#### Encapsulation efficiency of nano bio-fertilizer

A 5 mg of each nano capsule suspension solution trail was centrifuged at 6000 rpm for 30 min to separate the nano capsules from the aqueous medium containing the non-associated encapsulated materials. The suspension was left for 48 h in the dark under constant agitation to allow all entrapped active NPK nutrients to be available in the supernatant solution,where the intial percentage were 36.9%, 53.1%, 76.1% of N, P, and K, alternatively. The free materials in the supernatant were filtered with 0.2 µm membrane and vortexed for 10 s. Then, each NPK nutrient was determined using inductively coupled plasma mass spectrometer (ICP-MS) analysis for potassium, phosphorous ions, and CFU analysis for nitrogen ions. All trials were carried out in triplicate^23,24^. The encapsulation efficiency (EE%) of the nanoparticles was calculated according to the following equation:

2 $$\left( {{\text{EE}}\% } \right) \, = \frac{Total \;amount\; of\; NPK \;loaded - Free \;amount \;of\; NPK }{{Total \;amount \;of \;NPK \;loaded}} \times 100$$

#### Controlled release of active nutrients from nano bio-fertilizer

To investigate the controlled release of NPK nano fertilizer behavior from synthesized nano capsules, 50 mg of each lyophilized trail sample nano capsules were transferred to a dialysis bag and then immersed in 100 mL citrate phosphate buffer solution with (pH 5.5). At certain intervals, 10 mL of the solution was taken for NPK concentration determination, and another 10 mL of citrate phosphate buffer solution was added into the beaker to maintain a constant initial solvent volume. The samples were kept at 30 °C under gentle stirring at periodic intervals. These measurements were carried out in triplicate, and the data represent the average of these measurements. The amount of phosphorous and potassium ions concentrations were measured using ICP-MS, and nitrogen ions concentrations were measured using CFU analysis^23,24^.

The percentage of accumulative controlled release was calculated according to the following equation:

3 $${\text{E }} = \frac{{V_{E} \mathop \sum \nolimits_{1}^{n - 1} C_{i} + V_{0} C_{n} }}{{m_{o} }} \times 100$$

where E is the percentage of accumulative controlled Release (%). V_E_ is the volume of sampling volume (mL). V_0_ is the initial volume of the medium buffer (mL). C_i_, Cn is the concentration of NPK nano fertilizer (mg/mL) for initial and at a certain time. M_o_ is the mass of the sample taken for measurement (mg).

#### Water retention behavior of soil with and without formulated nano bio-fertilizer

This experiment was carried out to investigate soil's water retention behavior for formulated tested nano bio-fertilizers. A plastic cup containing 100 g of loamy sand soil and 10 g of lyophilized sample was mixed for both synthesized nano capsule trial samples. 50 mL of distilled water was poured, and the plastic cup was weighed as (W_0_). For the control trials, a plastic cup containing 110 g of loamy sand soil and 50 mL of distilled water was poured, and the plastic cup was weighed as (W_0_). In another reference of a plastic cup containing only 100 g of loamy sand soil without any sample, 50 mL of distilled water was added too, and the plastic cup was weighted as (W_1_) and weighted and set as a reference each time.

All plastic cups were kept in a lab room with ambient room temperature and weighed daily as (Wt) over 30 days with 5 days intervals.

The water retention percent as (WR %) of soil was determined using the following equation:

4 $$\left( {{\text{ WR}}\% } \right) \, = \frac{{W_{{t - W_{1} }} }}{{W_{{0 - W_{1} }} }} \times 100$$

#### Particle size and zeta potential analysis

Particle size and zeta potential of nano bio fertilizer analysis were obtained by dynamic light scattering (DLS) and Malvern nano series zeta sizer instrument. A 200 µg of each lyophilized tested sample was suspended in 2 mL of 0.2 µm filtered water, allowing total dissolution. Then, it was vortexed for 20 s and sonicated for 15 min. After that, the sample was filtered through a mciroyn glass vacuum filter with (10 µm particle retention) and sonicated for 5 min to dissolve fine nanoparticles fully. Subsequently, a small portion of the sample was placed in a plastic cuvette and carried to the analyzer chamber. All analysis measurements were performed at a scattering angle of 90 °C, temperature of 25 °C, and refractive index of 1.590. For each trial, the collective 24 reading was the mean diameter of particle size and zeta potential with a standard deviation of 100 iterations^23,24^.

#### Thermogravametric analysis

A (~ 20 mg) of each lyophilized sample was added into aluminum pans with a hole in the lid, and scanning was completed at a rate of 10 °C min^−1^ from 0 to 700 °C with N_2_ purging and measured with Pt crucible in N_2_gas flow (20 mL min^−1^) with a heating rate of 5 °C in the 25–900 °C range. The program was managed by the STAR software v 10.00 (Mettler Toledo).

#### Enumeration of P. fluorescence in the nano capsules

To estimate the viable counts of bacterial strain in the formulated nano capsules and the amount of viable bacterial strains without any contamination by measuring the releases encapsulated bacterial strain by resuspending 100 mg of nano capsules in phosphate-buffered saline (pH 7.0) for 40 min followed by homogenization. The total number of released bacterial strains was determined by the standard plate count method after incubating at 30 °C for 48 h. At every monthly interval, the cell densities in the nano capsules were enumerated using a similar method to study the cell loss upon storage^26^.

## Results and discussion

### Nano bio-fertilizer capsules

#### Synthesis and characterization of nano capsules

Nano bio-fertilizers are pivotal in modern agriculture, presenting a revolutionary approach to enhance nutrient availability, soil fertility, and overall crop productivity sustainability. Combining the strengths of nano fertilizer and bio-fertilizer technologies will offer innovative solutions with targeted nutrient delivery alongside beneficial microorganisms, ensuring sustained release at nano-sized encapsulated materials and improved uptake efficiency. Besides that, the benefit of bio-fertilizer techniques is that they shield the encapsulated microorganism from environmental stressors, enabling controlled release over time. Embracing nano bio-fertilizers can be a viable alternative to conventional agro-fertilizers and pesticides, effectively reducing nutrient loss through leaching and volatilization, thereby minimizing environmental pollution. The adoption of nano bio-fertilizers presents immense potential in addressing environmental resource security challenges while promoting eco-friendly farming practices, where this cutting-edge field represents a crucial area for ongoing research and development in agriculture.

**Figure 2:**
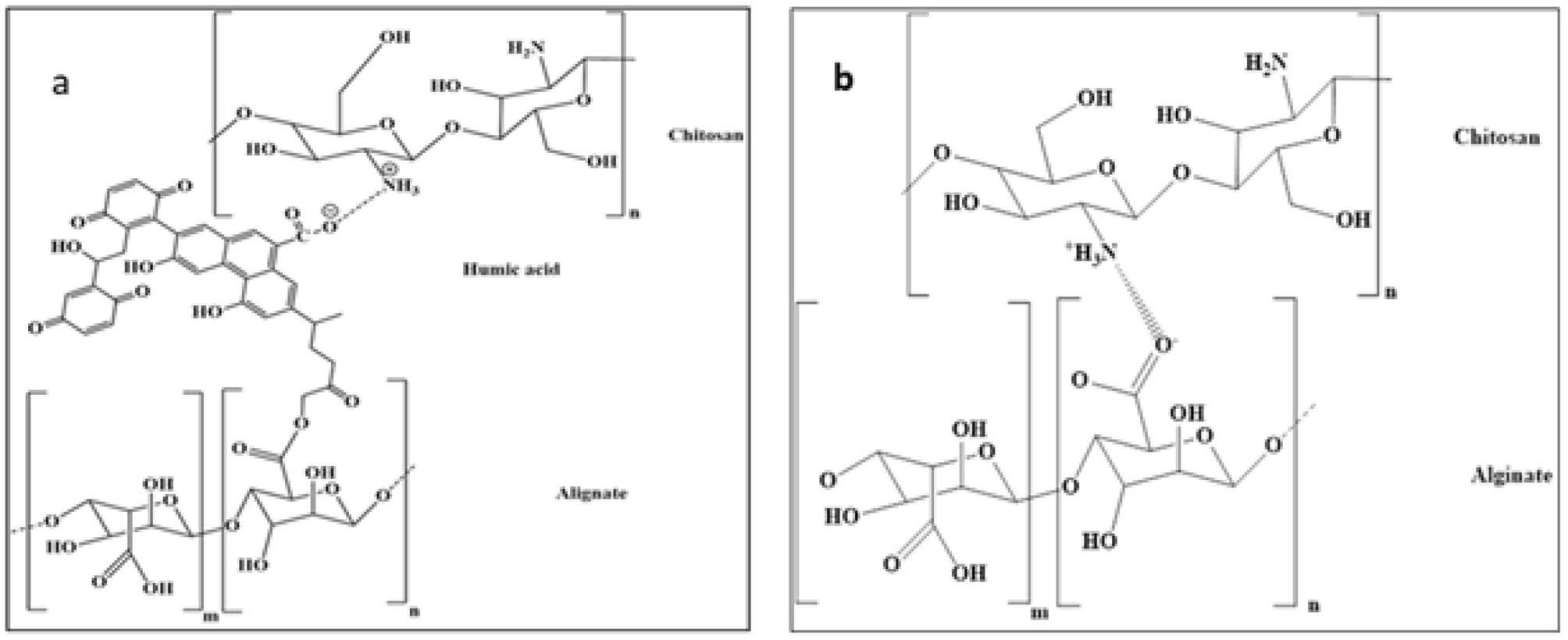
(**a**) Suggested (Ch./Alg.HA.NPK.PGPR) nano capsules interaction, (**b**) suggested (Ch./Alg. NPK.PGPR) nano capsules interaction.

This research synthesized novel nano bio-fertilizer capsules (Ch./Alg.) cross-linking with humic acid. These nanocapsules were loaded with essential agro-nutrients (NPK) and beneficial microorganisms from the (*P. Fluorescence*) strain, all at the nanoscale level. These novel capsules are referred to as (Ch./Alg.HA.NPK) or (Ch./Alg.HA.NPK.PGPR) nanocapsules. The diagrammatic representation of their proposed interaction is shown in Fig. 2. For comparative analysis, we involve Fig. 2a, b, another controlled nanocapsules of (Ch./Alg.NPK.PGPR) or (Ch.Alg.NPK) nanocapsules, without cross-linking agents. This was done to elucidate the impact of cross-linking on the stability of the nanocapsules and the controlled release of the entrapped materials.

In this study, the synthesized nano capsules were created using the ionotropic gelation polyelectrolyte method (IG-PEC). This method aimed to prepare nanoparticles that could encapsulate nano scale-sized active (NPK) fertilizer alongside beneficial microorganisms (*P. Fluorescence*). The process involved coating NPK fertilizer and *(P. Fluorescence*) with chitosan and alginate nanoparticle polysaccharides through cross-linking with humic acid in an aqueous solution. This humic acid cross-linking brought about several improvements: enhancing nano capsule stability, providing a suitable environment for selected PGPRs strain maintenance, and promoting adhesion between the two coating interfaces, making it more rigid and stable. This resulted in the highest potential payload of active (NPK) fertilizer and beneficial microorganisms (*P. Fluorescence)* at the smallest scale with a prolonged release effect to the targeted site, as illustrated in the following sections. These newly developed nano bio-fertilizer capsules have the potential to address numerous challenges in the agriculture sector and promote environmentally friendly crop production practices. Besides that, it reduces the reliance on traditional agrochemical fertilizers, offering a controlled and efficient method for delivering and releasing active (NPK) fertilizer and valuable materials.

Optimizing processing parameters of the prepared nano capsules of chitosan/alginate cross-linked with humic acid and its IG-PEC method has been adopted with the most stable mass ratio formulation, smallest particle size, surface charge, and highest encapsulation efficacy (EE). The particle size, zeta potential, and encapsulation efficacy %(EE) for control (Ch./Alg. NPK) nano capsule and (Ch./Alg. HA.NPK) nano capsule were measured to investigate the cross-linking effect on improving the nano capsule particle size, potential surface charge and encapsulation efficacy. The particle size and zeta potential of nano capsules play a crucial role in nanoparticle suspension’s stability to increase the encapsulation of its targeted materials. Nano capsules with a 50–500 nm diameter have been proven to have the best nano capsules properties^27^. The size distribution of nanoparticles depends on the molecular weight, concentration, and ratio of chitosan with other polymers and the cross–linking agents. The sign of zeta potential with ζ-potential more than − 30 mV dictates the stable main ionic charge of the nano capsules' surface. As shown in Fig. S.1. The (Ch./Alg. NPK) nano capsules show an average particle size of (316.3 ± 1 d.nm), an average ζ-potential of (− 24 ± 1.4 mV), and an average (EE) of (65 ± 0.99)%. The (Ch./Alg. HA.NPK) nanocapsules show an average particle size of (450.9 ± 0.530 d.nm), an average ζ-potential of (− 33 ± 0.95 mV), and an average (EE) of (87 ± 1.2)%. The (Ch./Alg. NPK) nano capsules show smaller average particle size, higher average zeta potential charge value, and lower encapsulation efficacy percentage than (Ch./Alg. HA. NPK) nano capsules.

The results of (Ch./Alg. NPK) nano capsules reveal that these particular nanocapsules exhibit limited stability and are prone to sedimentation over a defined timeframe, which was further validated through observations of settlement in approximately one month. Meanwhile, their particle size can be ascribed to amino groups within chitosan and hydroxyl alginate in stoichiometric ratios conducive to nano capsule formation. Nonetheless, the reduced encapsulation efficacy can be attributed to the preferential presence of NPK-negative ions, such as NO_3_^−^ and PO_4_^3−^, on the surface of the nanoparticles rather than being predominantly encapsulated within the capsules^28^.

This is also manifested in the elevation of the zeta potential value, which is intricately linked to the prevailing van der Waals interactions between the polysaccharide components, chitosan, and alginate, surpassing the influence of electrostatic repulsion in the ionotropic gelation polyelectrolyte method (IG-PEC) employed for preparation. In this context, chitosan typically assumes a dispersed arrangement due to the repulsion from its positively charged amino NH_3_^+^ groups in the polymer structure. Consequently, this structural disposition enhances the adsorption capacity for negatively charged ions, particularly the active NPK materials, onto the nanoparticles' surface. This interaction, in turn, exerts a direct impact on the stability of the nanocapsules.

**Figure 3:**
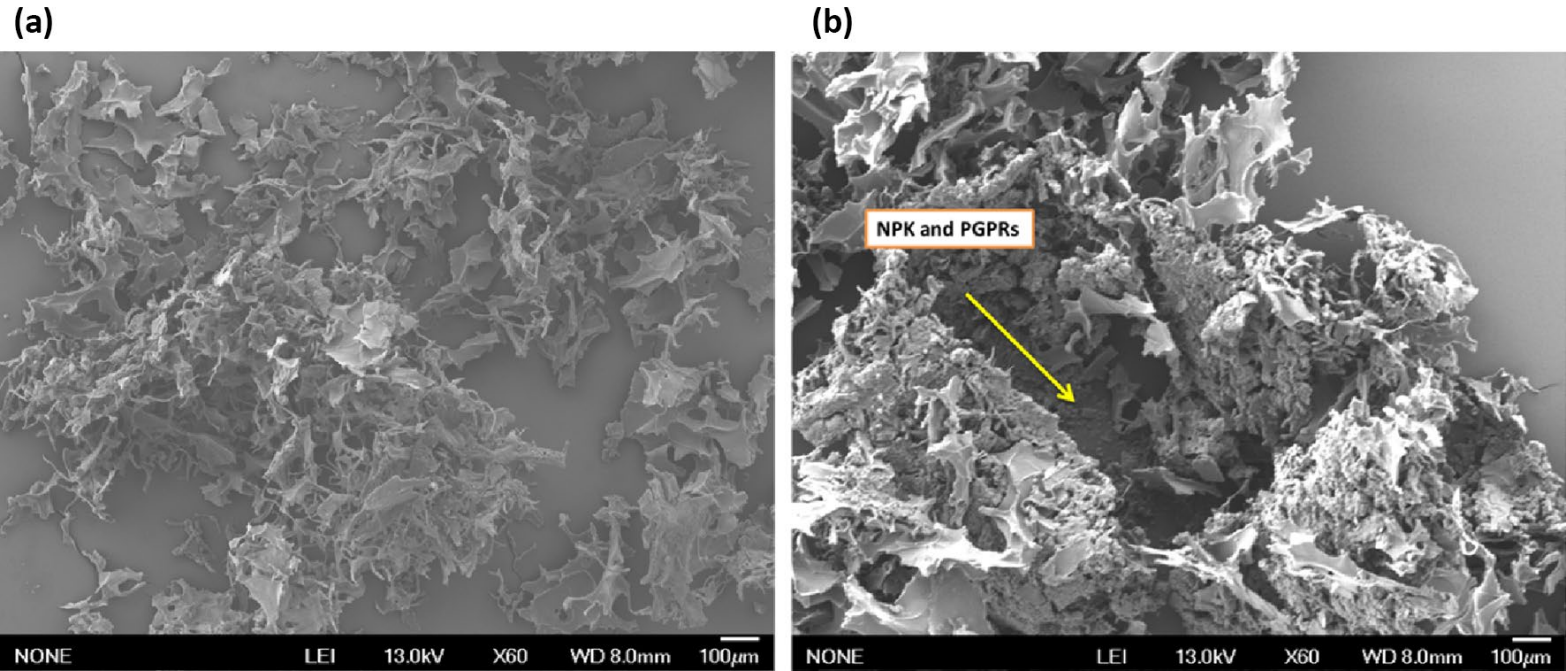
(**a**) Surface of (Ch. Alg.NPK.PGPRs) Nano capsule, (**b**) inside the matrix of nanocapsule with the appearance of entrapped NPK and PGPRs.

Meanwhile, the (Ch./Alg.HA.NPK) nanocapsules show larger particle sizes, higher encapsulation efficacy, and higher zeta potential value. The larger particle size can be ascribed to the role of humic acid in the formation of the organized network and plays a crosslinker upon formulating the nanocapsules in the ionotropic gelation polyelectrolyte method (IG-PEC) preparation method, where humic acid increased the availability of negatively charged hydrophilic carboxylate groups in the solution, which means more active carboxylate groups are ready to be linked with amino groups of chitosan polymer, as illustrated in Fig. 3. Consequently, the electrostatic interaction between chitosan and alginate materials will be the dominance over van der Waals interaction by entrapping more NPK fertilizer at the nano scale in its internal matrix, producing more extensive and less dense particles with strong repellent forces and higher encapsulation efficacy percentage. Furthermore, the heightened zeta potential exhibited by these nanocapsules plays a pivotal role in determining the potential allocation of chitosan within the dispersion particles. Notably, humic acid demonstrates a preferential orientation towards alginate, as opposed to chitosan, effectively bolstering the stability of the interfacial matrix. Notably, the NPK fertilizer assumed an ionic configuration, specifically NO_3_^−^ and PO_4_^3−^ ions. This particular ionic composition could potentially lead to interactions between these ion groups and the internal matrix constituted by humic acid and alginate, with minimal absorption occurring on the surface of the nanocapsules. Consequently, the NPK fertilizer entrapped within the emerging internal network modulates the surface charge, enhancing encapsulation efficiency. This phenomenon indicates that the formulated nano fertilizer possesses the essential attributes requisite for effective utilization in agronomic contexts.

#### Surface structure and morphology analysis

**Figure 4:**
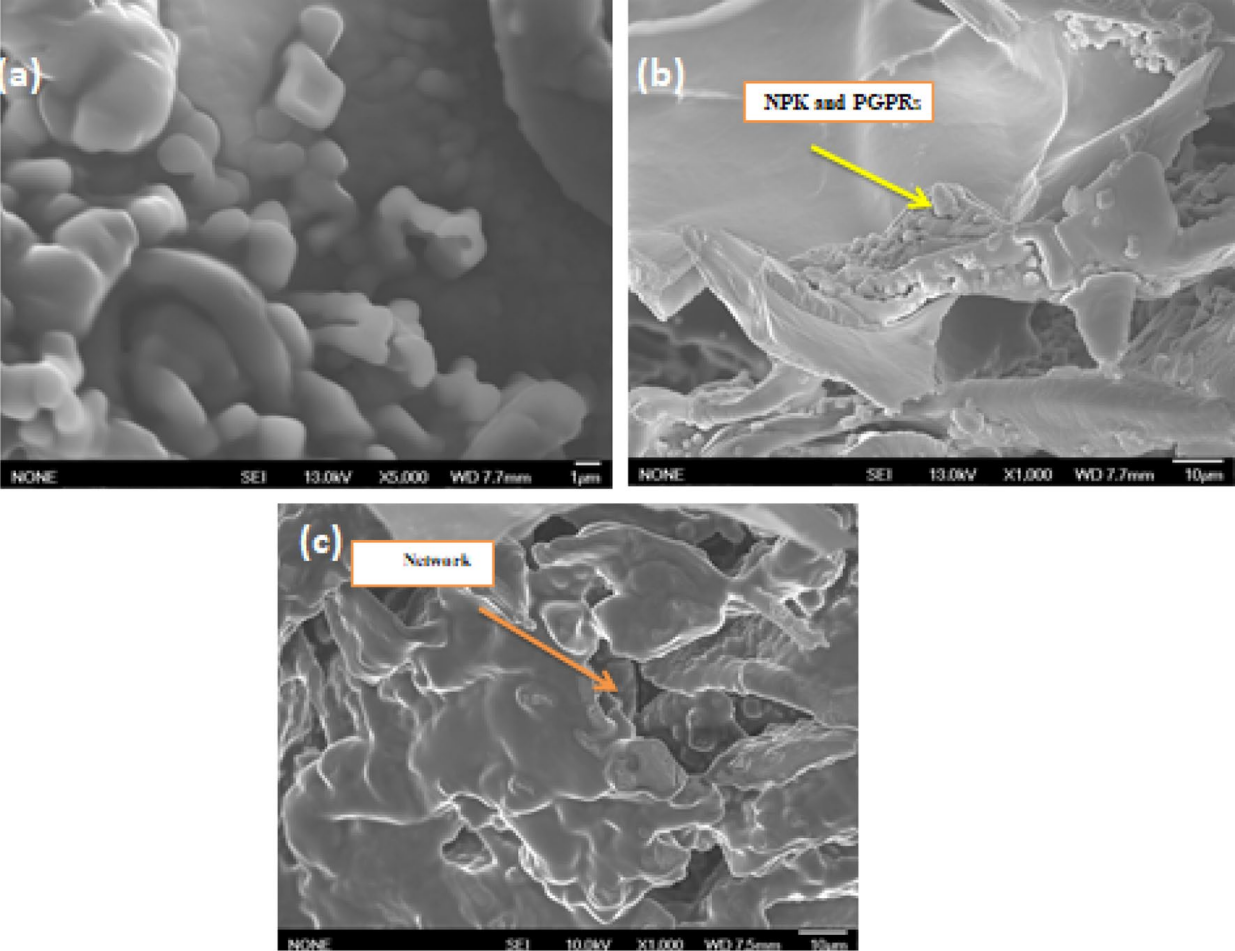
(**a**) The surface appearance of (Ch. Alg.HA.NPK. PGPRs), (**b**) inside the matrix of nanocapsule with the appearance of entrapped NPK and PGPRs, (**c**) network of cross-linking inside the nanocapsule with embedded entrapped materials.

The surface structure and morphologies of (Ch./Alg.NPK.PGPRs) nanocapsules and (Ch./Alg.HA.NPK.PGPRs) nanocapsules were shown in Figs. 3 and 4. They have been studied using the scanning electron microscope (SEM) technique. Figure 3a and b show alternatively the surface structure and morphologies for (Ch./Alg.NPK.PGPRs) nanocapsule As shown in Fig. 3a, the (Ch./Alg.NPK.PGPRs) nanocapsules were not of distinct spherical shape; they appear less physically stable and seem to have porosity and hollowness.

Meanwhile, Fig. 4a–c (Ch./Alg./HA.NPK.PGPRs) most resembled a spherical shape with a spherical shell and core type regular arrangement, as well as appeared to be smooth in its shell appearance, which means chitosan was able to sufficiently coat these nanoparticles with efficient loading of NPK materials and PGPRs strain.

This change in morphology between (Ch./Alg./HA. NPK.PGPR) and (Ch./Alg. NPK.PGPRs) nanocapsules can be attributed to the incorporation of alginate with chitosan and its cross-linking effect with humic acid, where the existence of amorphous products with interconnected porous network able to increases with the presence of active ingredients to be distributed on contact surface area and within the hydrogel network pores of formulated nanocapsules, leading to more active nutrient absorption in the preparation method. Also, it should be noteworthy that the interlinked pore of the humic acid, as cross-linked, can delay the dissolution of the loaded entrapped targeted materials as revealed in the release profile and result in the slow release mechanism behavior**.**

#### FT-IR spectral analysis

Fourier transform infrared spectroscopy (FTIR) is a powerful analytical technique for analyzing various materials' molecular composition and structure.

**Figure 5:**
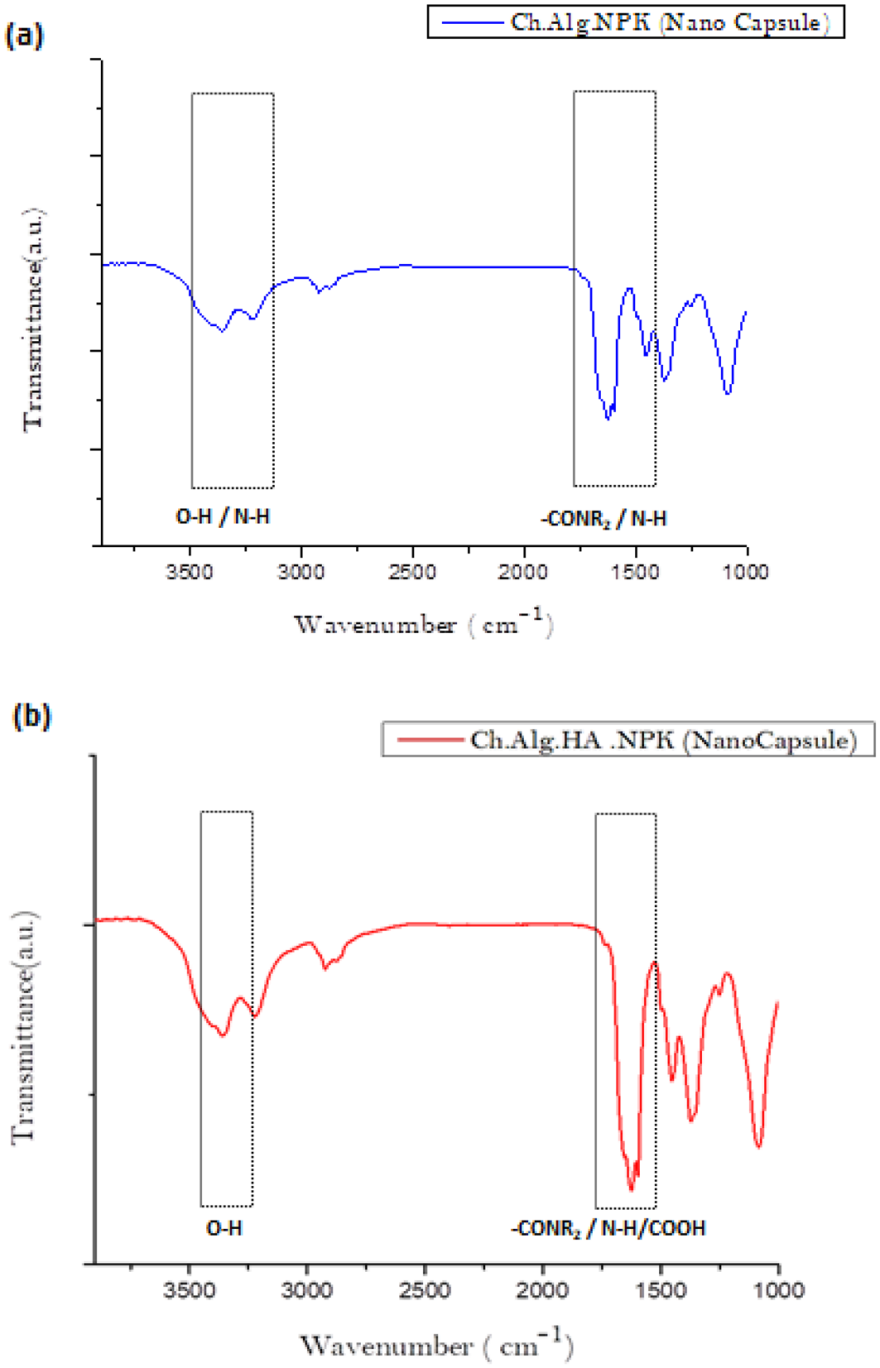
(**a**, **b**) IR of (Ch.Alg.NPK), (Ch.Alg.HA. NPK) nanocapsules.

Figure 5a, b shows the FT-IR spectra of (Ch. Alg. NPK) and (Ch. Alg. HA. NPK), as the H.A. indicates noticeable change as a cross-linked fertilizer formulation proceeds to completion of nanocapsules formation.

Its FT-IR spectra results are similar to those obtained in other studies for chitosan polymer, as shown in Fig. S.2.a. The chitosan spectrum shows firm functional group peaks at 3368 cm^−1^ and 656 cm^−1^ corresponding to the N–H and O–H stretching frequency of glucosamine and intermolecular hydrogen bonding. Significant peaks at 2885 and 2900 cm^−1^ are allocated to C–H symmetric and asymmetric stretching. These bands are the main characteristics of typical chitosan polysaccharides. Other peaks at 1651 cm^−1^ and 1546 cm^−1^ correspond to the C–O stretch in the primary alcohol group. The absorption peak at 1376 cm^−1^ is allotted to C–N glucose stretching of primary amine, while those at 1404 cm^−1^ and 1424 cm^−1^ are allotted to CH_2_ and CH_3_ symmetrical deformation, respectively.

Alginate is a linear polymer derived from brown seaweeds. As shown in Fig. S.2.b. The FTIR study of alginate provides information on its molecular structure and functional groups. Alginate, like chitosan, has distinct peaks in the FTIR spectrum: the carbonyl (C=O) stretching vibration of the carboxyl groups in alginate corresponds to the peak at approximately 1566 cm^−1^. The asymmetric stretching vibration of the alginate carboxylate group (COO^−^) corresponds to the peak at 1411 cm^−1^. The peak shows the stretching vibration of the C–O bond in the carboxylate group at 1249 cm^−1^. The peak at around 1249 cm^−1^ corresponds to the stretching vibration of the C–O bond in the carboxylate group. The peak at about 1095 cm^−1^ corresponds to the stretching vibration of the C–O–C bond in alginate.

The resulting spectrum obtained from the FTIR analysis of humic acid in Fig. S.2.c. will show characteristic absorption peaks corresponding to different functional groups in the sample. Some standard absorption bands observed in the FTIR spectrum of humic acid include carboxylic acids with a peak around 1700 cm^−1^, indicating the presence of carboxylic acid groups (–COOH). The peak bands around 1650 cm^−1^ indicate the presence of aromatic rings, and the peak at 2930 cm^−1^ indicates the presence of aliphatic hydrocarbon chains. The hydroxyl (–OH) group peak is around 3350 cm^−1^.

Figure 5a of (Ch. Alg.NPK) nanocapsule gives the FT-IR analysis of the chitosan-alginate complex, revealing a shift in the amide carbonyl group to 1628 cm^−1^ and a shift in the chitosan amino group to 1566 cm^−1^, indicating the interaction between chitosan and alginate.

Meanwhile, Fig. 5b of (Ch.Alg.HA) nanocapsule shows that combining chitosan with alginate and humic acid creates new interactions and compatibility between these components. FTIR analysis aids in understanding these interactions and probable chemical reactions by studying changes in functional groups. The FT-IR analysis of the Chitosan with Alginate and Humic Acid complex revealed a shift in the amide carbonyl group to 1651 cm^−1^, a shift in the chitosan amino group to 1542 cm^−1^, a shift in carboxylic acid groups (–COOH) to 1734 cm^−1^, and shift in the hydroxyl (–OH) groups to 3420 cm^−1^. These variations can be attributed to hydrogen bonding, electrostatic interactions, or other types of chemical bonding.

In summary, FTIR analysis of chitosan, alginate, and chitosan with alginate and humic acid provides precise information about their molecular composition, functional groups, and possible interactions. Researchers can analyze the FTIR spectra to gain insights into these materials' structural properties and chemical behavior, which is valuable for agricultural purposes.

#### Thermogravametric analysis

**Figure 6:**
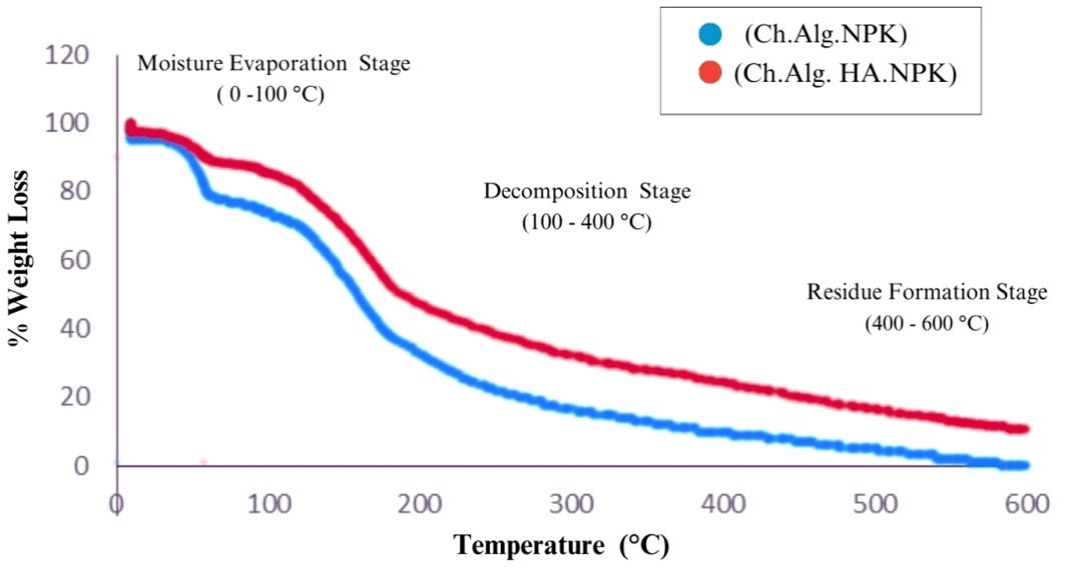
TGA analysis for (Ch. Alg. NPK) and (Ch. Alg. HA.NPK) nanocapsules.

TGA is a thermal analysis technique that analyzes the thermal behavior of materials as their temperature changes. TGA can offer significant information about thermal stability, breakdown behavior, and compatibility, as shown in Fig. 6 for (Ch. Alg.NPK) and (Ch. Alg. HA.NPK) nanocapsules.

Change to following:

TGA analysis of (Ch. Alg. NPK) nanocapsules entails submitting the sample to a controlled temperature increase and measuring the accompanying weight changes. The TGA curve derived often exhibits the following significant features:

- Weight loss at first: The TGA curve may show a minor reduction in weight at lower temperatures due to the evaporation of moisture adsorbed on the sample's surface.
- Primary decomposition stage: Chitosan-alginate polymers are thermally decomposed in a multi-step process. The TGA curve typically displays a significant weight loss during the main breakdown stage. The polymeric backbone of chitosan and alginate degrades, yielding volatile degradation products such as carbon dioxide, carbon monoxide, and water vapor. The study shows that alginate-chitosan composite can experience weight losses ranging from 30 to 85.7% when subjected to temperatures between 100 and 400 °C.
- Residue formation: A residue may remain after the major breakdown step, often consisting of inorganic components or burned material. This residue's weight reveals information about the thermal stability of the leftover alginate fragments and the presence of contaminants.

TGA analysis of (Ch.Alg. HA.NPK) nanocapsule provides information about the thermal behavior of the composite and any interactions among the components. The TGA curve for the alginate-chitosan-humic acid composite may resemble the following extra features:

- Interaction and compatibility: Compared to the above analysis, the TGA curve may reveal differences in breakdown behavior and heat stability. The study shows that (Ch. Alg. HA.NPK) nanocapsule composite can experience weight losses ranging from 10 to 61.1% when subjected to temperatures between 100 and 400 °C. This suggests that alginate, chitosan, and humic acid interact via hydrogen bonding and chemical interactions.
- Synergistic effects: Chitosan and Humic acid can alter the thermal stability of the composite. Synergistic effects, in which the combination of components improves overall thermal stability, can be noticed as a greater decomposition temperature or lower weight loss than the other nanocapsule.
- Residue formation: The composition of the residue following thermal degradation may differ in the composite. The residue may contain chitosan, humic acid, and any leftover alginate fragments.

#### Water retention behavior of nano capsules in soil

**Figure 7:**
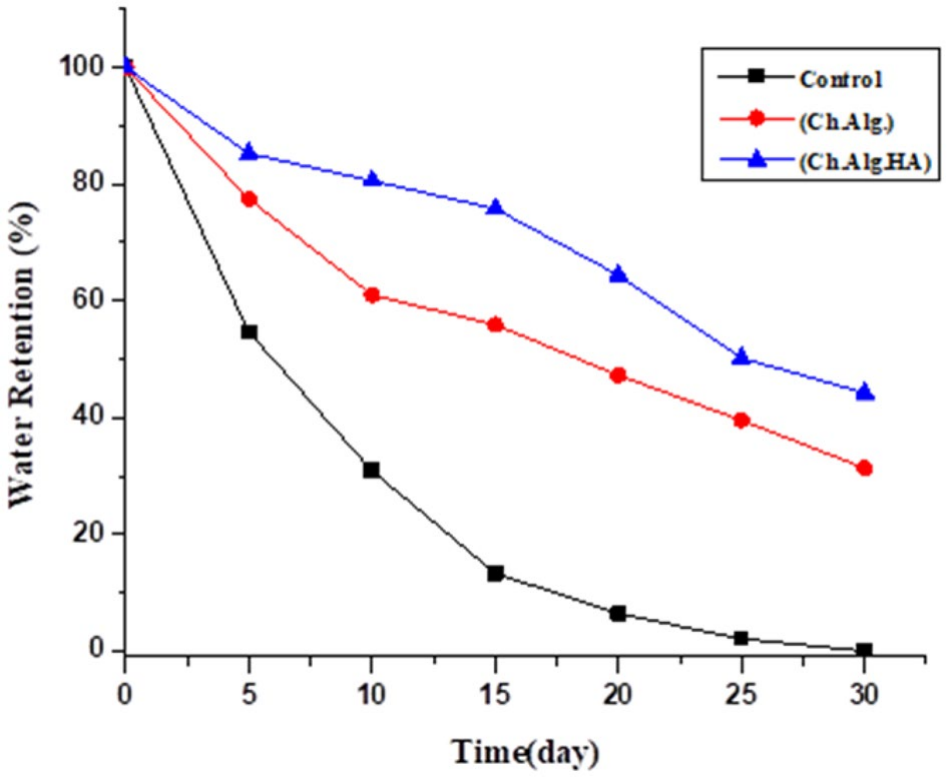
Water retention of formulated (Ch.Alg.NPK.PGPRs) and (Ch. Alg. HA.NPK.PGPRs) nano capsules.

The water retention behavior of synthesized (Ch. Alg. NPK) and (Ch. Alg. HA.NPK) nano capsule**s** were studied in loamy sand soil, as shown in Fig. 7 and Table 1.

**Table 1:** Water retention percentage for (Ch.Alg.NPK) and (Ch. Alg. HA. NPK) Nanocapsules.

Time	Control		Ch.Alg		Ch.Alg.HA		Weight (ref)
Days	Weight (Wt)	Percentage	Weight (Wt)	Percentage	Weight (Wt)	Percentage	
0	160	100	160	100	160	100	150
5	152.739	54.62	156.4	77.5	157.648	85.3	144
10	141.218	30.95	149.39	61	154.723	80.6	132.8
15	124.816	13.34	142.26	55.9	150.175	75.8	119.4
20	114.136	6.4	134.128	47.2	142.556	64.4	111
25	106.155	2.1	126.78	39.6	132.72	50.2	105
30	100	0	118.84	31.4	126.52	44.2	100

Notably, the prepared (Ch./Alg. HA.NPK.PGPRs) nanocapsules increased the water retention percentage with 44.2% of the soil more than the (Ch. Alg. NPK.PGPRs) nanocapsules of 31.4% for the studied period. These findings revealed that humic acid with the hydrophilic carboxylate groups that cross-linked and incorporated with chitosan and alginate nanoparticles in the hydrogel network of formulation enhances the absorption of water molecules inside the nanocapsules and preserves it for a more extended period, and thus improve the water retention behavior. As a result, using these nano bio fertilizer capsules in farming practices can decrease irrigation frequencies and enhance its sustained and prolonged release.

#### Nano bio-fertilizer release studies

**Figure 8:**
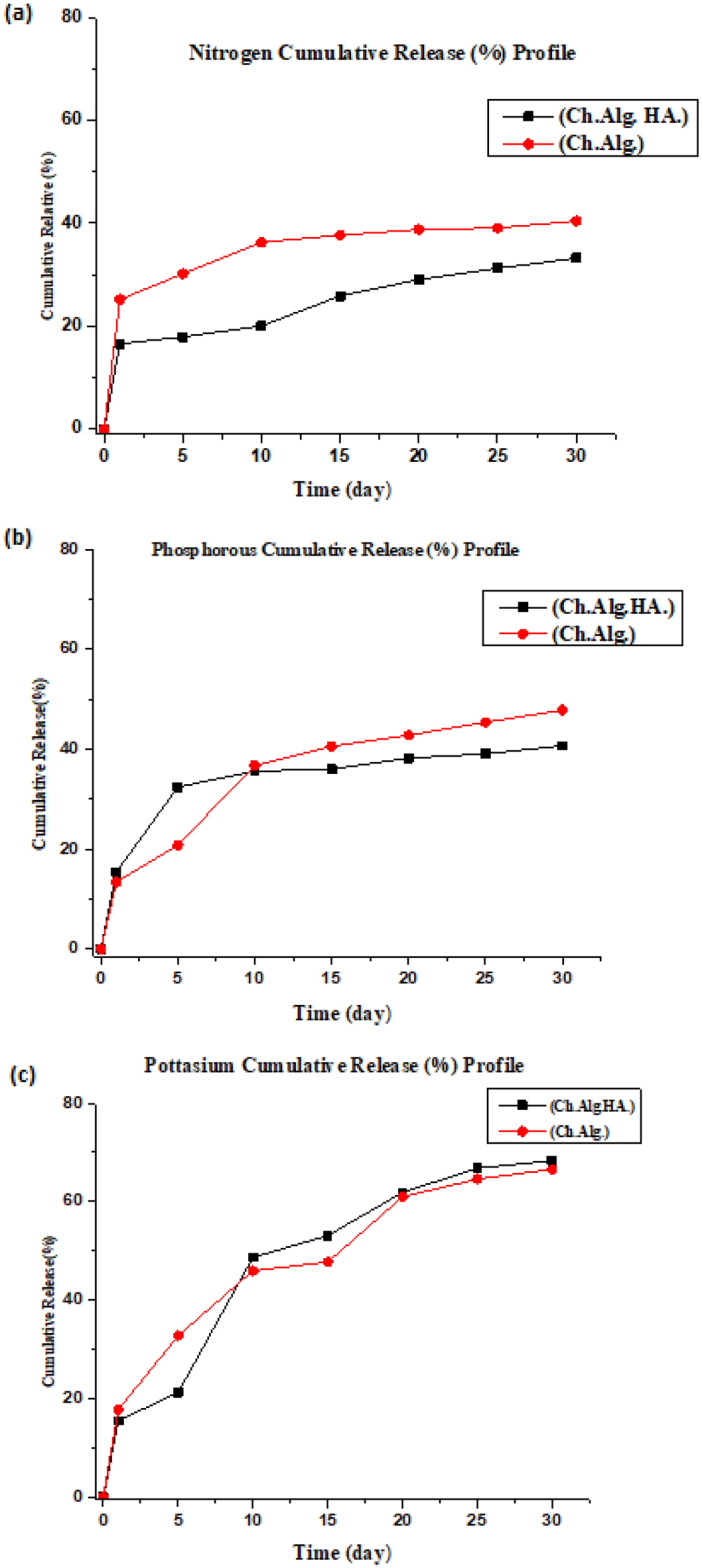
(**a**) Nitrogen cumulative release (%) profile for synthesized nanocapsules, (**b**) phosphorous cumulative release (%) profile for synthesized nanocapsules, (**c**) potassium cumulative release (%) profile for synthesized nanocapsules**.**

The cumulative release profile for synthesized nanocapsules formulation was performed as a function of time in pH solution and is presented in Fig. 8a–c and Tables 2, 3, 4 for Nitrogen, Phosphorous, and Potassium, respectively. The controlled cumulative release profile is defined as the rate of active substances released from the nanocapsules, where the nanocapsules can control the release of entrapped target materials and provide multiple functional benefits; usually, the nanocapsules are made of polysaccharide materials with particular barrier properties for manipulating and controlling the release of entrapped materials.

**Table 2:** Release behavior of active nutrient Nitrogen for (Ch.Alg.NPK) and (Ch. Alg. HA. NPK) Nanocapsules.

Time	Log time	(Ch.Alg.HA) (mg ml^−1^)	% Cumulative release	Log (Mt/Mo)	(Ch.Alg.) (mg ml^−1^)	% Cumulative release	Log (Mt/Mo)
0	0	0	0	0	0	0	0
1	0	0.028	6.6	0.8195	0.069	15.18	1.181
5	0.7	0.079	17.94	1.253	0.131	30.2	1.480
10	1	0.084	20.06	1.302	0.153	36.28	1.559
15	1.2	0.110	25.88	1.412	0.157	37.7	1.576
20	1.3	0.122	29.04	1.463	0.162	38.78	1.588
25	1.4	0.131	31.26	1.494	0.163	39.1	1.59
30	1.5	0.139	33.2	1.521	0.169	40.44	1.606

**Table 3:** Release behavior of active nutrient phosphorous for (Ch.Alg.NPK) and (Ch. Alg. HA. NPK) nanocapsules.

Time	Log time	(Ch.Alg.HA) mg ml^−1^	% Cumulative release	Log (Mt/Mo)	(Ch.Alg.) mg ml^−1^	% Cumulative release	Log (Mt/Mo)
0	0	0	0	0	0	0	0
1	0	0.070	15.4	1.187	0.061	13.42	1.1277
5	0.7	0.141	32.42	1.510	0.089	20.8	1.318
10	1	0.149	35.6	1.551	0.159	36.76	1.565
15	1.2	0.150	35.98	1.5558	0.170	40.58	1.608
20	1.3	0.160	38.2	1.582	0.179	42.78	1.631
25	1.4	0.163	39.06	1.591	0.190	45.38	1.656
30	1.5	0.170	40.66	1.609	0.200	47.8	1.679

**Table 4:** Release behavior of active nutrient potassium for (Ch.Alg.NPK) and (Ch. Alg. HA. NPK) nanocapsules.

Time	Log time	(Ch.Alg.HA) mg ml^−1^	% Cumulative release	Log (M_t_/M_o_)	(Ch.Alg.) mg ml^−1^	% Cumulative release	Log (M_t_/M_o_)
0	0	0	0	0	0	0	0
1	0	0.073	15.46	1.189	0.087	17.74	1.248
5	0.7	0.090	21.26	1.327	0.141	32.76	1.51
10	1	0.213	48.66	1.687	0.187	45.96	1.66
15	1.2	0.222	53.1	1.725	0.200	47.74	1.678
20	1.3	0.261	61.86	1.791	0.259	60.98	1.78
25	1.4	0.280	66.82	1.824	0.270	64.58	1.81
30	1.5	0.285	68.3	1.834	0.278	66.56	1.823

The controlled release profile can be classified into two modes: sustained or delayed. The sustained release is designed as the release of entrapped materials in constant release with maintaining concentration to the target site. The delayed release is designed to delay the release of entrapped materials to a point where the release is required. The release of active NPK nutrients and bacterial strains in this research will be initiated by using a buffered solution stressor with (a pH of 5.5) to stimulate the soil media^29^.

Implementing the release profile in vitro conditions assures the nanocapsules' functionality and the release behavior of entrapped materials in actual agriculture applications with similar environmental conditions. Also, it can validate the type of diffusion release mechanisms as a cumulative release or percentage release of encapsulated materials at a particular time.

The release profile of these active NPK nutrients and bacterial strains has shown to undergo two phases within this stressed buffered solution. The first phase is about the initial burst release of encapsulated materials, and the second phase is the uniform controlled release in a sustained manner. This effect occurs when water is absorbed into the nanocapsules, where the large concentration of entrapped active NPK materials will be attached near the surface of the particle membrane, allowing it to undergo the first phase of rapid release^29,30^.

After that, the second phase occurred with the diffusion of entrapped active nutrients from the inner compartment and core in the first-period intervals. Then, over time, the nanocapsules degraded to release the entrapped active NPK nutrients (Fig. 8a–c) and bacterial strain, where the release involves three steps: absorption of the solution into a loaded nanocapsule system, swelling of the inner matrix, conversion of the glassy polysaccharide polymer into a ruby matrix, and finally diffusion of the active nutrient and microorganism from the rubbery matrix, which is faster than matrix erosion^29,30^.

The chitosan polymer membrane acts as a barrier to control the release of active substances, and the diffusion mechanism limits the diffusion of active substances by controlling their diffusion from its location in the matrix to the surface of the nanocapsules. Mainly, the Diffusion mechanism is controlled by several factors, such as temperature, the solubility of nanocapsules and their permeability, and the vapor pressure effect of the membrane.

Slow-release kinetics of the active NPK nutrients were analyzed for both prepared nano bio-fertilizer capsule trials by immersing it in the stressed buffered (pH 5.5) solution. The result of N, P, and K slow release from the nano bio-fertilizer capsules is shown in Fig. 8a–c, respectively, where two curves belong to (Ch. Alg. HA.NPK) nanocapsules and (Ch. Alg..NPK) nanocapsules^30,31^.

Changed to this one:

Figure 8a shows the cumulative nitrogen release profile of (Ch./Alg. HA.NPK) nanocapsules show a preferable sustained release behavior in a controlled manner compared to the curve of the (Ch./Alg..NPK) nanocapsules; this can be related to the role of humic acid which worked a barrier effect in releasing the entrapped components. It is shown that ((Ch./Alg. HA.NPK) nanocapsules cumulative nitrogen release profile was approximately 33.2% within 30 days compared to (Ch./Alg.NPK), which was about 40.44% within the same study period. These results indicate the efficient role of humic acid in controlling the release of ionic nitrogen compounds and altering its release to be sustained with prolonged effect.

Figure 8b shows the cumulative phosphorous release profile of (Ch./Alg. HA.NPK) nanocapsules also shows a preferable sustained release behavior in a controlled manner compared to the curve of the (Ch./Alg..NPK) nanocapsules; this can be related to the role of humic acid which worked a barrier effect in releasing the entrapped components. It is shown that ((Ch./Alg. HA.NPK) nanocapsules cumulative phosphorous release profile was approximately 40.7% within 30 days compared to (Ch./Alg.NPK), which was about 47.8% within the same study period.

Figure 8c shows the cumulative potassium release profile of (Ch./Alg. HA.NPK) nanocapsules and preferable sustained release behavior in a controlled manner compared to the curve of the (Ch./Alg..NPK) nanocapsules. It is shown that ((Ch./Alg. HA.NPK) nanocapsules cumulative potassium n release profile was approximately 68.3% within 30 days compared to (Ch./Alg.NPK), which was about 66.56% within the same study period. It is noticed that the cumulative release of (Ch. Alg. NPK) was less than (Ch. Alg. HA.NPK). This may be attributed to the small size of potassium ions and the ease of release of larger nanocapsule sizes from formulated pores in its network; also, the positive charge enhances its release where there is no interaction with the chitosan surface.

On the other hand, the curves showed that nitrogen nutrient was slowly released compared to other phosphorous and potassium nutrients.

The slow release of nitrogen can be explained by the ionic bond force between chitosan nanoparticles on the surface with the negative nitrogen ions when it reaches the surface at the diffusion phase. In contrast to potassium ions, they inhibited the fast release and were followed by phosphorous ions, which may also be affected by the formulation of ionic bonds formed between these ions and chitosan nanoparticles on the surface. However, these ionic bonds seemed less stable than nitrogen ionic bonds, which are visualized in their cumulative release profile^32^.

On the other hand, the number of bacterial strain inoculums was also measured, and it was found that the number of high viability bacterial cells slightly decreased to 1.7 × 10^10^ CFU g^−1^ with minimum cell loss; this indicated that nanocapsules were capable of releasing cells for more than six months.

Also, The kinetic release for all cumulative releases of entrapped active nutrients for both synthesized nanocapsules was analyzed by Korsmeyer and Peppas module as shown in Tables 2, 3, 4^33^. The release profile for each nutrient was fitted into the Korsemeyer peppas model Eq. (7) as follows:

7 $$\frac{{M_{t} }}{{M_{0} }} = {\text{K}}_{{{\text{kp t}}^{{\text{n}}} }}$$

where $\frac{{M_{t} }}{{M_{0} }}$ = is the released fraction of active nutrients at time t. K_kp_ = is a constant incorporating characteristics of nanocapsule. (n) = diffusional exponent, as indicative of the transport mechanism.

The linearization of all graphs for all nutrients for both synthesized nanocapsules samples generates an equation of the line: y–the axis is the log of cumulative release, and the x-axis is the log of time. The linear equation shows the slope as the release exponent(n) as the release mechanism, and the y-intercept is log release and its rate constant^34^. Suppose the diffusion exponent (n) value for the release of fertilizer is n ≤ 0.5. In that case, it means that the active nutrient release mechanism approaches a Fickin diffusion-controlled sustained release module, whereas if the n value is equal to n = 1, it indicates that the active nutrient release approaches the zero-order mechanism transport. Meanwhile, if the n value is between 0.5 and 1, it shows that active nutrient release is anomalous non-Fickin diffusion.

It can be seen from Tables 2, 3, 4, that all NPK nutrients have a release mechanism (n) that lies between 0.4 and 0.8; the nitrogen diffusional exponent for (Ch.Alg.HA. NPK) was approximately 0.5, which means it follows the Fickin diffusion-controlled sustained release module; whereas, the (Ch. Alg. NPK) 's nitrogen release diffusional exponent (n) was approximately 0.6, which means the nitrogen release is anomalous non-Fickin diffusion module.

For the phosphorous release, it shows that the diffusional exponent(n) for (Ch. Alg.HA. NPK) and (Ch.Alg.NPK) nanocapsules is also approximately equal to 0.5, which means both nanocapsules release follow the Fickin diffusion-controlled sustained release module.

Finally, for the potassium kinetic release, diffusional exponent (n) intersects with their release behavior, and the highest cumulative release for both synthesized nanocapsules is approximately equal to 0.7, whose transport mechanism is a non-Fickin diffusion module.

This diffusional exponent of all active nutrients for both synthesized nanocapsules indicates controlled and matrix-based releases^35^. Besides that, the high value of the y-intercept supports the profile that release undergoes stress-solution conditions.

The prepared (Ch. Alg. HA.NPK.PGPRs) nanocapsule in this research work exhibits several advantages compared with the controlled synthesized (Ch. Alg. NPK. PGPRs) nanocapsule. The one-step preparation process of this novel nano-bio fertilizer loaded with active nutrients and beneficial microorganisms by a simple in-situ polymerization technique can significantly reduce the production cost of these prepared nanocapsule formulations. Using a humic acid molecule not only advances the cross-linking forming between chitosan and alginate polysaccharides but embeds a high amount of encapsulated material, enhancing the water absorption capacity and providing a release property.

Finally, using such nano bio-fertilizer products not only can be an alternative to conventional agrochemical fertilizers but can also increase the quality and quantity of agricultural products by improving crop fertility and soil water conservation.

## Conclusion

This research focuses on the utilization of chitosan in the synthesis of innovative nano bio-fertilizer capsules, aiming to deliver active agro nutrients, namely Nitrogen, Phosphorous, and Potassium ions (NPK), along with beneficial plant growth-promoting rhizobacteria (PGPRs) microorganisms. These entrapped components are coated using innovative cross-linking of chitosan and alginate with humic acid through ionic gelation and polyelectrolyte complexation technique. The synthesized (Ch.Alg.HA.NPK.PGPRs) nanocapsules show resembled spherical shape nanocapsules with an interconnected porous network regular type arrangement with a particle size of (450.9 d.nm.), ζ-potential of (− 33 mV), and encapsulation efficiency (87%). The thermal stability of these nanocapsules shows a percentage mass residuals of more than 25% at 600 °C, indicating these capsules’ stability for an efficient time in soil. Besides, its water retention capability percentage was 44.2%. The initial percentage of each of N, P, and K were in synthesized nanobio fertilizer sample as 36.9%, 53.1%, and 76.1%, alternatively, and the release test behavior of entrapped materials shows a cumulative release percentage of N, P, and K ions to be 33.2%, 47.8%, and 68.3%, alternatively within 30 days. Also, the release mechanism using the kinetic module of the Korsemeyer- Peppas mathematical model shows a Fickin diffusion controlled release module governs the release of N and P ions with a diffusional exponent = 0.5, and the non-Fickin diffusional module governs the release of K ion with a diffusional exponent = 0.7. The findings provide insights into implementing nano bio-fertilizers to efficiently deliver agro-nutrients, which can contribute to agricultural productivity and sustainability.

### Supplementary Information

Supplementary Figures.

## Data Availability

Adequate and clear descriptions of the applied materials and tools are provided in the materials and method section of the manuscript. In addition, the obtained data is justifed by mentioning the figures and tables in the manuscript.

## Abbreviations

PGPRs: Plant growth promoting rhizobacteria
NPK: Nitrogen-Phosphorous-Potassium
ACC: Amino cyclopropane-1-carboxylic acid
Auxin: Indoleacetic acid
P. Fluorescence: Pseudomonas fluorescence
IG: Inotropic gelation
PEC: Polyelectrolyte complexation
ROS: Reactive oxygen species
CNDs: Carbon nano dots
CQDs: Carbon quantum dots
IAA: Indole acetic acid
Ch.ZnO: Chitosan.ZnO
Ch. CNDs: Chitosan.CNDs
Ch. Hybrid: Chitosan. Hybrid
MIC: Minimum inhibitory concentration
CFU: Colonies forming unit reduction
Ch. Alg. NPK.PGPRs: Chitosan. Alginate. NPK.PGPRs Nano capsules
Ch. Alg. HA. NPK.PGPRs: Chitosan. Alginate. Humic Acid. NPK. PGPRs
Val.Ch.: L- Valine. Chitosam
Tryp.Ch.: L-Tryptophan. Chitosan
Val. Ch.Lys.: L- Valine. Chitosan. L- Lysine
Tryp.Ch.Lys.: L- Tryptophan.Chitosan.L-Lysine
PAM: Pulse amplitude modulated
