# Supplementary material for: Nano bio fertilizer capsules for sustainable agriculture
Source: Sci Rep. 2024 Jun 13;14:13646. doi: 10.1038/s41598-024-62973-5 (PMC11176377; doi:10.1038/s41598-024-62973-5)

**Figure. S.1.** The DLS (particle size) measurements of synthesized Nano capsules


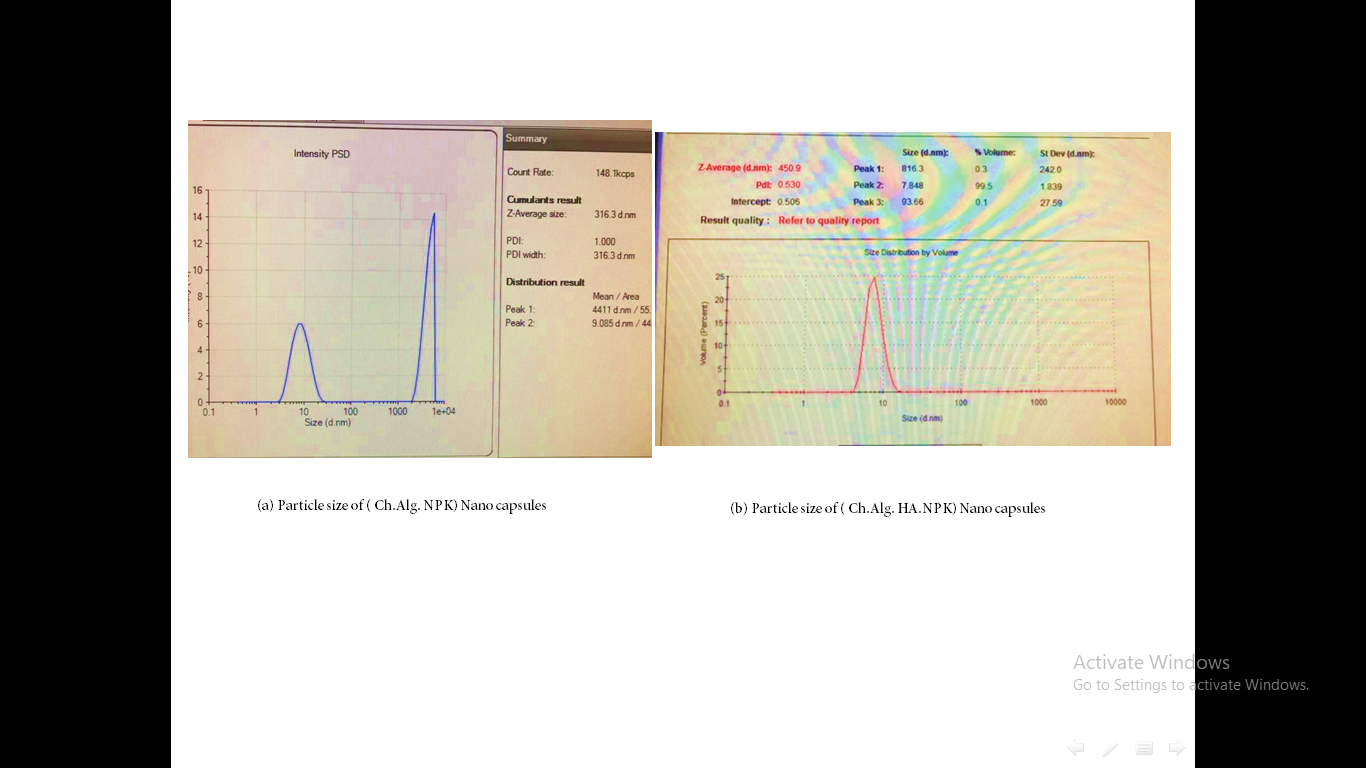


Figure S.2. FT_IR of Chitosan , Alginate , Humic Acid


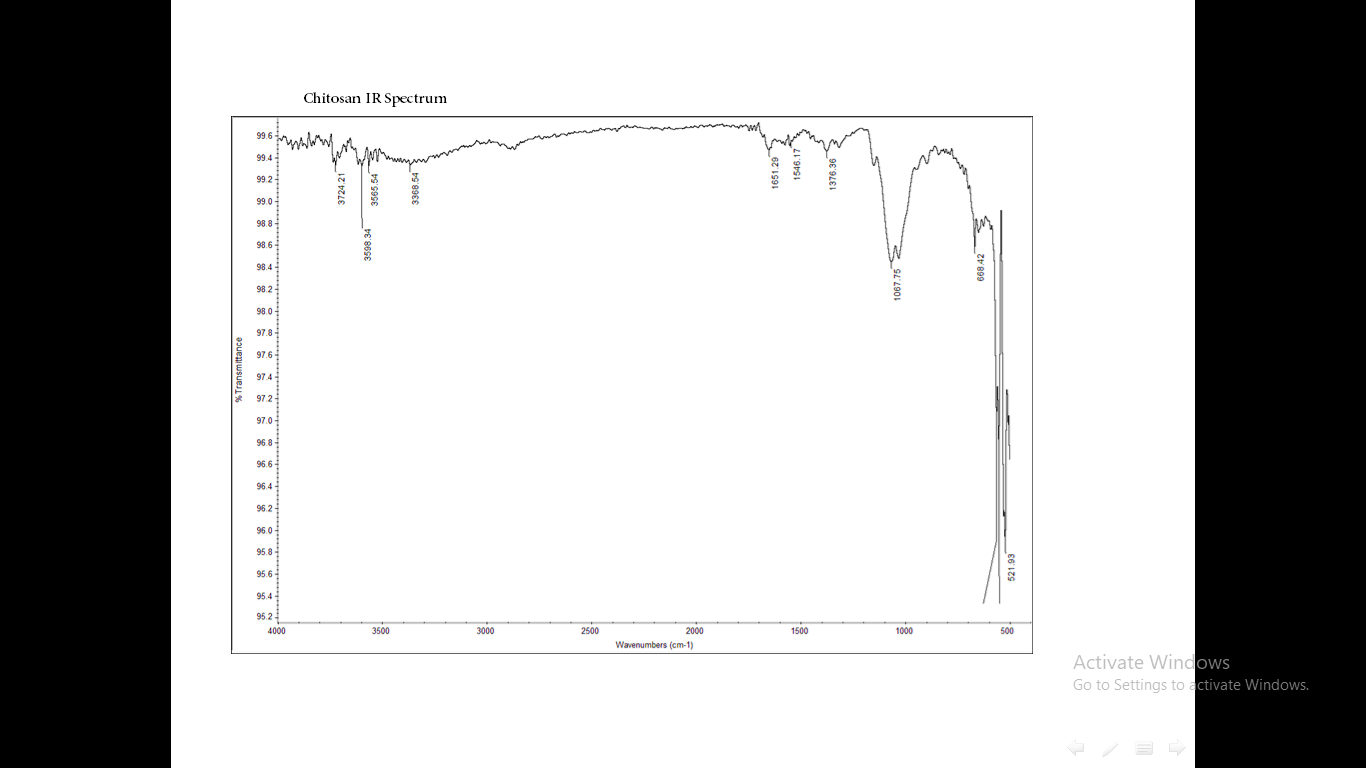


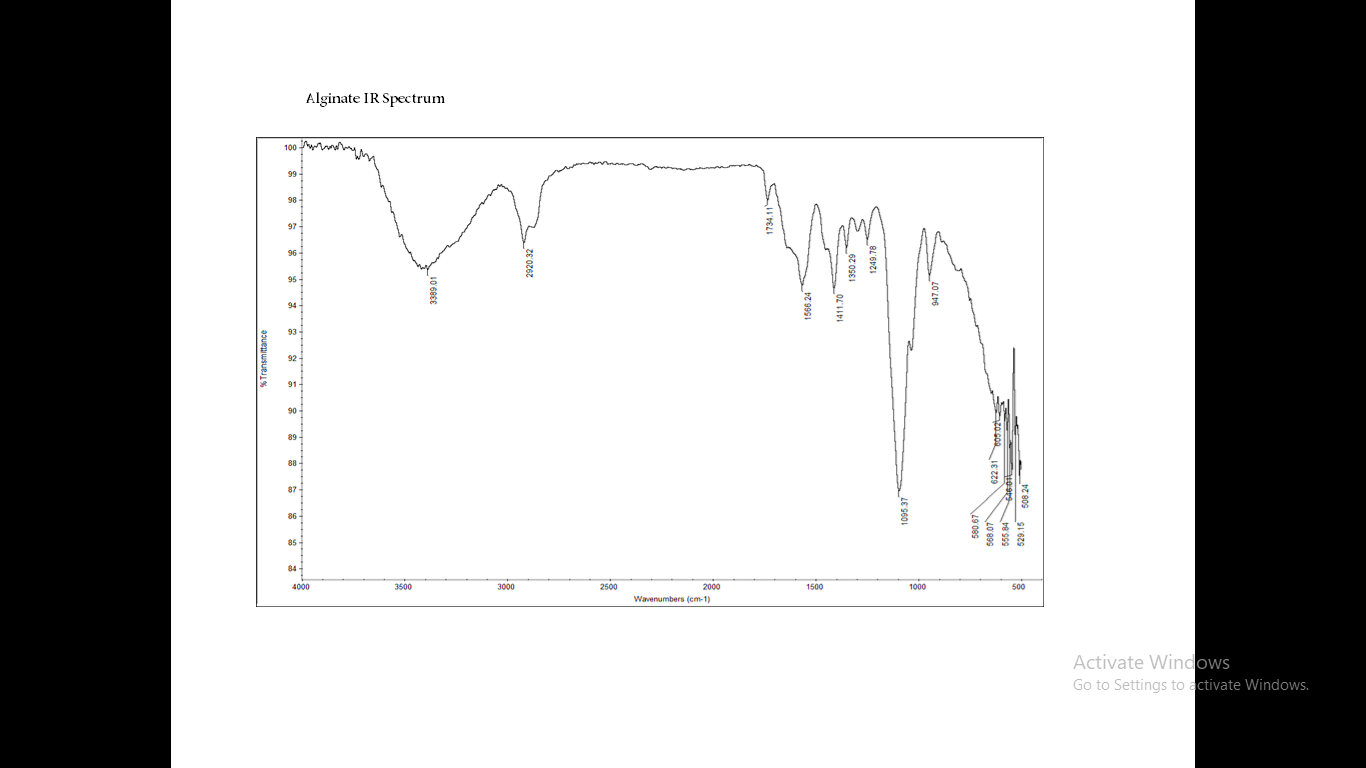


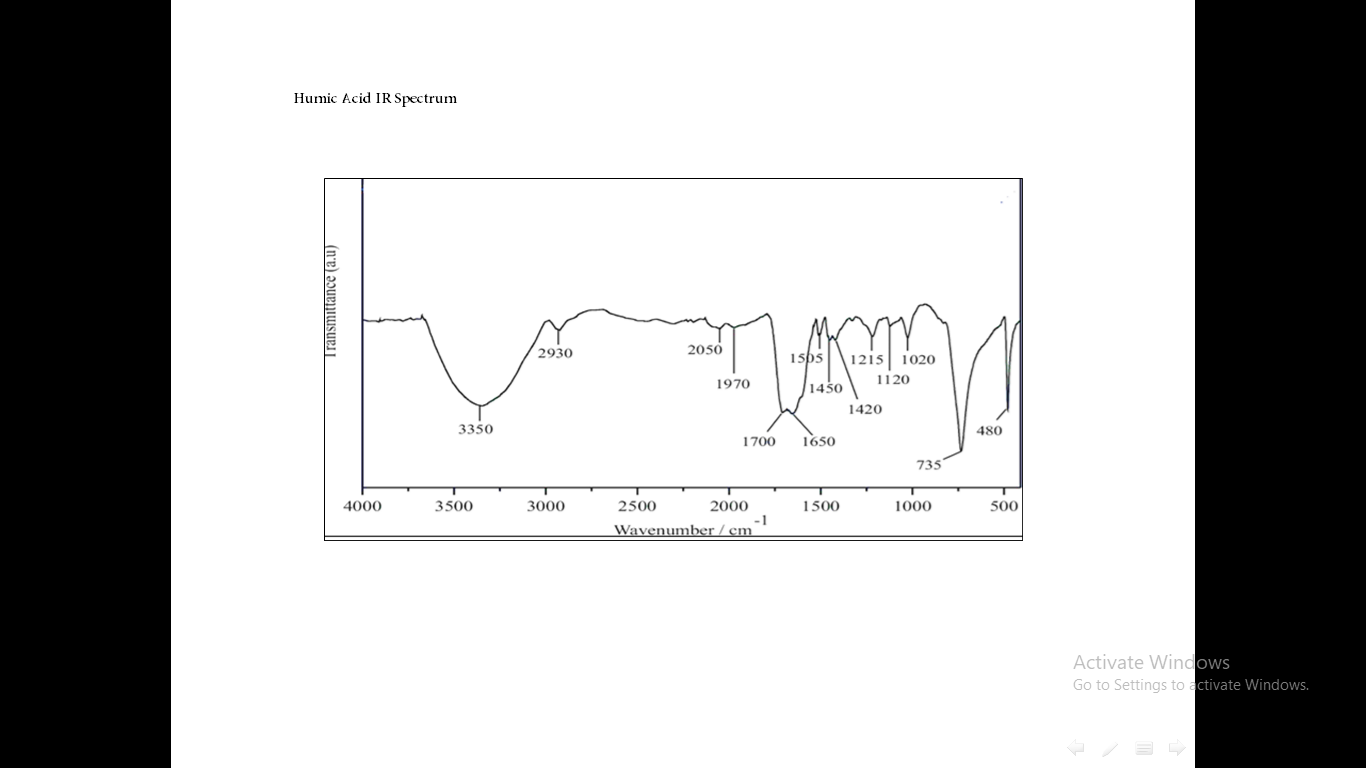

Supplement: Supplementary file 1 — Supplementary Figures. [file 41598_2024_62973_MOESM1_ESM.docx]
